# Evaluating psychological distress associated with life events under the traumatic experience threshold in patients with major depressive and bipolar disorder

**DOI:** 10.1038/s41598-024-67101-x

**Published:** 2024-07-15

**Authors:** Hiroki Ishii, Tasuku Hashimoto, Aiko Sato, Mami Tanaka, Ryota Seki, Michi Ogawa, Atsushi Kimura, Michiko Nakazato, Masaomi Iyo

**Affiliations:** 1 Department of Psychiatry, Graduate School of Medicine, Chiba University, 1-8-1 Inohana, Chuo-ku, Chiba, 260-8670 Japan; 2 Department of Psychiatry, International University of Health and Welfare Narita Hospital, 852, Hatakeda, Narita, 286-8520 Japan; 3 Division of Clinical Study on Juvenile Delinquency, Center for Forensic Mental Health, Chiba University, 1-8-1 Inohana, Chuo-ku, Chiba, 260-8670 Japan; 4 Department of Psychology, Faculty of Liberal Arts, Teikyo University, 359 Otsuka, 192-0395 Hachioji, Japan; 5 Department of Psychiatry, Chiba Hospital, 2-508 Hasamacho, Funabashi, 274-0822 Japan

**Keywords:** Psychology, Psychiatric disorders

## Abstract

Patients with bipolar disorder (BD) and major depressive disorder (MDD) experience psychological distress associated with daily events that do not meet the threshold for traumatic experiences, referred to as event-related psychological distress (ERPD). Recently, we developed an assessment tool for ERPD, the ERPD-24. This tool considers four factors of ERPD: feelings of revenge, rumination, self-denial, and mental paralysis. We conducted a cross-sectional study between March 2021 and October 2022 to identify the differences and clinical features of ERPD among patients with MDD and BD and healthy subjects who did not experience traumatic events. Specifically, we assessed ERPD using the ERPD-24 and anxiety-related symptoms with the State-Trait Anxiety Inventory, Liebowitz Social Anxiety Scale, and anxious-depressive attack. Regarding the ERPD-24 scores among the groups, as the data did not rigorously follow the test of normality, the Kruskal–Wallis test was used to compare the differences among the groups, followed by the Dunn–Bonferroni adjusted post-hoc test. Non-remitted MDD patients and BD patients, regardless of remission/non-remission, presented more severe ERPD than healthy subjects. This study also demonstrated the relationships between all anxiety-related symptoms, including social phobia and anxious-depressive attack and ERPD, in both BD and MDD patients and in healthy subjects. In conclusion, patients with non-remitted MDD and with BD regardless of remission/non-remission experience severe ERPD related to anxiety-related symptoms.

## Introduction

It is well known that various life events can cause or trigger the onset, relapse, and recurrence of psychiatric disorders. Traumatic events (e.g., wars, disasters, dangerous accidents, abduction, harsh physical and sexual violence in domestic or intimate contexts) are the most serious. Such events can directly cause trauma-related disorders, such as acute stress disorder and post-traumatic stress disorder (PTSD) and constitute one of the diagnostic criteria for these disorders^1^. However, everyone experiences numerous stressful events in daily life that do not meet the threshold for traumatic experiences. These stressful events (e.g., interpersonal, health, and work-related problems) frequently trigger the onset and relapse of mood disorders^2,3^.

In previous studies, we found that non-remitted patients with major depressive disorder (MDD)^4^ and bipolar disorder (BD)^5^ experience more serious psychological distress associated with daily life events that do not qualify as traumatic experiences—based on Diagnostic Criterion A for PTSD in the DSM-5 and ICD-11^1,6^—compared to remitted patients. Given that everyone can experience non-traumatic life events, it is necessary to deeply understand the psychological distress associated with life events in patients with MDD and BD. Therefore, to precisely evaluate psychological distress associated with life events under the threshold for traumatic experiences, referred to as event-related psychological distress (ERPD), we developed an assessment tool, the ERPD-24^7^, which consists of four factors: feelings of revenge, rumination, self-denial, and mental paralysis^8^.

This study is the first to examine psychological distress related to daily life events under the traumatic experience threshold among patients with mood disorders using an assessment tool (ERPD-24) specifically designed for this purpose rather than an alternative trauma-specified assessment measure. The purpose of this study was to clarify the features of ERPD—including feelings of revenge, rumination, self-denial, and mental paralysis—in patients with MDD and BD using the ERPD-24 by comparing them to healthy subjects. Based on our previous studies^4,5^, we hypothesized that the severity of ERPD in non-remitted patients with MDD and BD would be higher than in remitted patients and healthy people. In addition, recent reports suggest that rumination-related negative thinking, which partly overlaps with the concept of ERPD, could be influenced by not only depression but also anxiety^9–11^ and autism^12^. Therefore, we also explored the relationships between ERPD and various clinical characteristics, such as anxiety-related symptoms, using appropriate measures.

## Methods

### Ethics statement

The study protocol was approved by the ethics committees of Chiba University Graduate School of Medicine (ID 4047), the International University of Health and Welfare, Chiba Psychiatric Medical Center, Chiba Hospital, Kokoronokaze Clinic Funabashi, and Sodegaura Satsukidai Hospital. All participants provided written informed consent for participation in this study after the procedure was fully explained to them. All the tests were performed in accordance with the Declaration of Helsinki.

### Participants and design

This was a cross-sectional study. Participants were recruited from among patients commuting to or hospitalized at Chiba University Hospital, International University of Health and Welfare Narita Hospital, Sodegaura Satsukidai Hospital, Chiba Psychiatric Medical Center, Chiba Hospital, and Kokoronokaze Clinic Funabashi. This study was conducted between March 2021 and October 2022. All patients voluntarily participated. Participants had to meet the following eligibility criteria: (1) aged 20–69 years and (2) no history of traumatic experiences that met the ICD-11^6^ and DSM-5 criteria for PTSD. Individuals who had no ERPD or ERPD-related life events were excluded by assessing their ERPD using the ERPD-24. We interviewed each in- and outpatient face-to-face in a consultation room at each facility for around 60 min.

To recruit patients, we surveyed available in- and outpatients at the research institutes. Patients were included if they were diagnosed with MDD or BD according to the DSM-5 criteria using the Japanese version of the Mini International Neuropsychiatric Interview (M.I.N.I.)^13,14^. Patients were excluded if they were diagnosed with PTSD, schizophrenia spectrum and other psychotic disorders, neurocognitive disorders (e.g., dementia, organic mental disorders, intellectual disabilities), or imminent suicidal ideation.

We recruited healthy control subjects with social networking ads. Potential control subjects were excluded if they had a history of or were currently diagnosed with psychiatric disorder based on the M.I.N.I., regularly took psychotropic medication, had first-degree relatives with psychiatric disorders, or were pregnant. We also interviewed each healthy subject face-to-face in a consultation room at each facility for around 40 min.

### Assessment of depression, mania, and remission

The severity of depression was assessed using the Quick Inventory of Depressive Symptomatology Self-Report, Japanese version (QIDS-J)^15,16^. We evaluated the severity of mania using the Young Mania Rating Scale (YMRS)^17^. Remission state was defined by a QIDS-J score ≤ 5 in patients with MDD and by both a QIDS-J score ≤ 5 and a YMRS score ≤ 7 in patients with BD, similar to euthymia. Depressive state was defined as a QIDS-J score > 5 and a YMRS score ≤ 7 in patients with MDD and BD. The manic/hypomanic state was defined as a YMRS score of > 7. We categorized patients who satisfied the diagnostic criteria for mixed features in the DSM-5 as having a QIDS-J score > 5 and a YMRS score > 7 in patients with BD and MDD.

### Assessment of ERPD associated with life events under the threshold for traumatic experiences

ERPD comprises psychological phenomena, such as flashbacks, resentment, and regret, related to events considered mundane, such as interpersonal problems and financial difficulties that fall short of experiences that qualify as traumatic under the diagnostic criteria for PTSD in both the DSM-5 and ICD-11. We used the ERPD-24—a self-administered scale that we developed—to assess the severity of the subjects’ ERPD in the week prior to and including the date of their response to our questionnaire^7^ (Version 1.0 of the ERPD-24 and its instruction manual are presented in the Supplemental File). As stated in the Introduction, the four factors comprising ERPD in the ERPD-24 include “feelings of revenge” (7 items), “rumination” (7 items), “self-denial” (6 items), and “mental paralysis” (4 items).

In our instructions for the ERPD-24, we advise that trauma survivors should be excluded using the diagnostic criteria for PTSD (DSM-5 and ICD-11). The instrument asks respondents to identify the events that still cause them to experience unpleasant memories and feelings and accordingly provide their answers for each item, to specify when the events occurred, how often they had recalled the events in the past month, and how long they have continued to remember the events. Regarding the scoring method, this scale uses a four-point Likert scale, ranging from 0 (not at all) to 3 (very much so). The scores for each option were added, and the scores for the entire scale and for each subscale were calculated. The higher the score for each scale, the higher the severity of ERPD.

### Categorizing life events related to ERPD

We classified the participants’ life events regarding ERPD into ten groups based on a list of threatening experiences^18^: separation from a close person, family problems that do not involve physical and sexual domestic violence or other forms of abuse, health-related problems, interpersonal problems, changes in living conditions, job-related events, sex-related events, neglect, bullying, and other events.

### Assessment of anxiety-related symptoms

To assess the severity of anxiety-related symptoms, including social phobia, we used the State-Trait Anxiety Inventory (STAI)^19^ and the Japanese version of the Liebowitz Social Anxiety Scale (LSAS)^20,21^.

Anxious-depressive attack (ADA) is a recently proposed symptom cluster consisting of sudden intense feelings of distressing emotions with no direct psychological cause^11^. ADA is characterized by intrusive rumination on mainly regretful memories accompanied by a violent emotional storm, resulting in countermeasures, including acting out. Based on the original criteria for ADA^11^, we also evaluated whether patients had ADA or not.

### Assessment of other psychological features

Following the evaluation of the ERPD-24, we assessed the Impact of Event Scale-Revised (IES-R)^22,23^ for the same events as ERPD in this study. We also assessed autistic traits using the Japanese version of the Autism Spectrum Quotient (AQ)^24,25^. The presence of autistic traits was defined as an AQ score of ≥ 33. Furthermore, among patients with MDD, we also surveyed whether those with bipolarity met the criteria of either bipolar spectrum disorder^26^ or bipolar specifiers^27^.

### Assessment of clinical characteristics

We surveyed demographic data, including age, sex, comorbidity, educational background, duration of disease, therapy duration, current employment, marital status, and family history of psychiatric diseases in first-degree relatives. Among patients with BD, we diagnosed those with bipolar I or II disorder using the M.I.N.I.

### Primary and secondary outcomes

The primary outcome of this study was to identify the differences in ERPD severity (based on the total and factor ERPD-24 scores) among patients with MDD and BD and healthy subjects. The secondary outcomes were to explore the detailed characteristics of ERPD among these groups and to examine the relationships between ERPD and clinical characteristics, such as anxiety symptoms, among these groups.

### Statistical analysis

According to the assessment of the mood states of depression, mania, and remission (euthymia in BD) outlined above, we classified the participants into the six following groups: depressive BD patients, manic/hypomanic BD patients, remitted BD patients, non-remitted (i.e., depressed) MDD patients, remitted MDD patients, and healthy subjects. All analyses were conducted using SPSS version 28 (IBM Corporation, Armonk, NY, USA). For categorical variables, the chi-square test was used. We used Student’s t-test to compare the two groups. We used the Kruskal–Wallis test for the other variables because the data regarding the ERPD in the subgroups did not rigorously follow the test of normality. To compare the differences among the groups, we used the Dunn–Bonferroni adjusted post-hoc test after the Kruskal–Wallis test. Pearson correlation tests were conducted to examine the correlations between the ERPD scores and other psychometric data. The 95% confidence interval (CI) was used to estimate precision, and a *p* < 0.05 was considered significant.

## Results

### Participants’ characteristics

**Figure 1:**
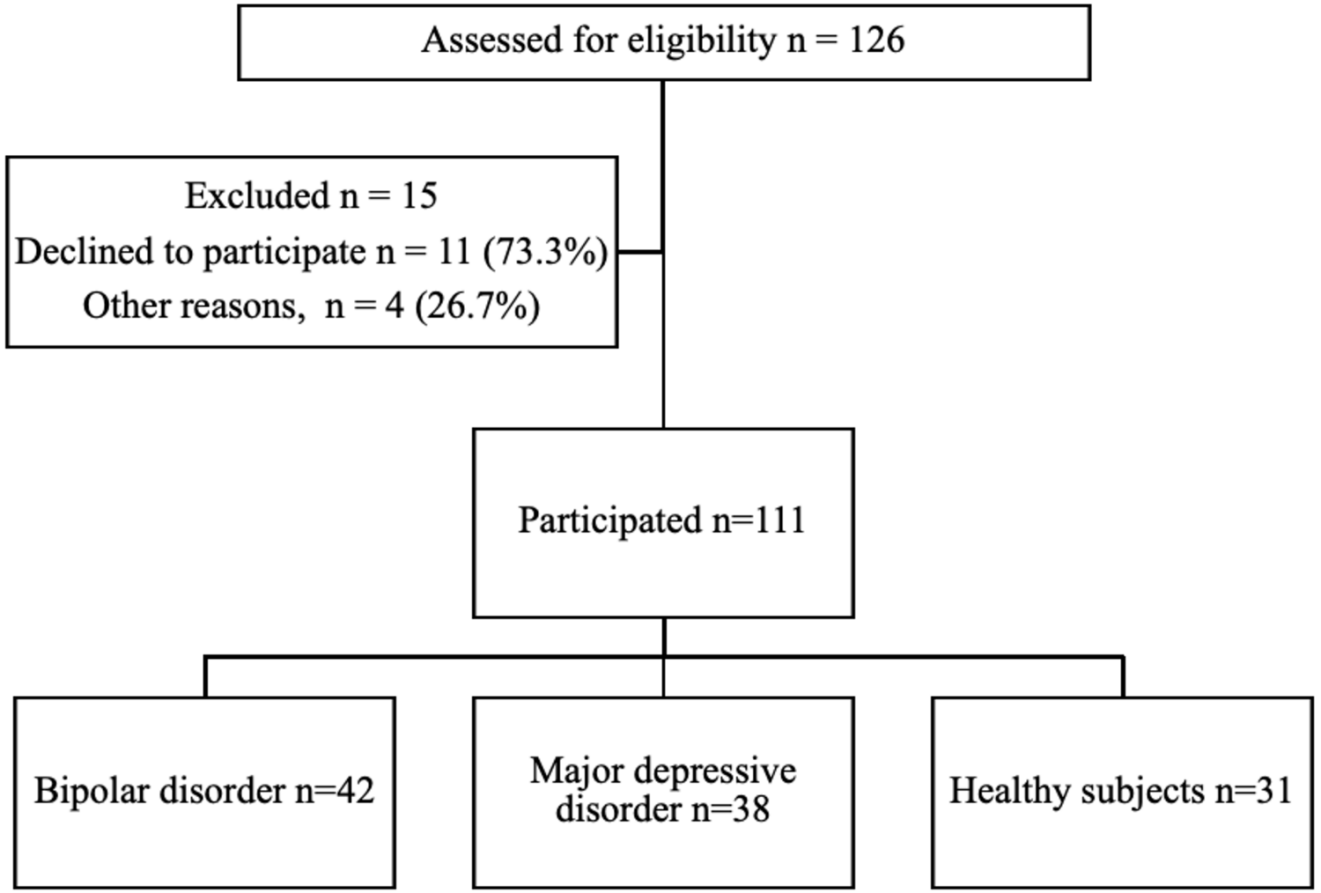
Study flowchart. Of the 126 patients screened, 111 were enrolled in this study.

Figure 1 shows a flowchart of eligible candidates and subjects who participated in this study. Ultimately, 95 patients with MDD and BD (76 outpatients and 19 inpatients) and 31 healthy volunteers were eligible to participate. Of these patients, 11 declined to participate and four were unable to complete the interview. Finally, 80 patients with MDD (n =38) and BD (n =42) and 31 healthy subjects were included. All participants reported that they had experienced at least one ERPD or ERPD-related event. The participants’ characteristics are shown in Table 1. Table 2 details the psychiatric comorbidities among the patients with BD and MDD.

**Table 1:** Characteristics of the patient groups and healthy subjects.

		Bipolar disorder			Major depressive disorder		Healthy subjects	*F*/χ^2^	*p*
		Depression	Mania/hypomania	Remission	Depression	Remission			
		(*n* = 19)	(*n* = 12)	(*n* = 11)	(*n* = 27)	(*n* = 11)	(*n* = 31)		
Age, mean (*SD*)		44.50 (12.00)	44.30 (14.50)	39.40 (14.40)	44.30 (12.70)	51.50 (12.90)	45.8 (13.90)	0.94	0.46
Sex, male		9	5	4	13	5	15	0.64	0.99
Female		10	7	7	14	6	16		
Outpatient/in-patient		12/7	8/4	11/0	23/4	10/1			
Highest level of school completed (%)									
Junior high school		2 (10.5)	0 (0.0)	2 (18.2)	0 (0.0)	0 (0.0)	0 (0.0)		
High school		5 (26.3)	4 (33.3)	1 (9.1)	11 (40.7)	3 (27.3)	5 (16.1)		
Vocational school/Junior college		3 (15.8)	8 (66.7)	3 (27.3)	3 (11.1)	4 (36.4)	12 (38.7)		
University		8 (42.1)	0 (0.0)	4 (36.4)	10 (37.0)	4 (36.4)	13 (41.9)		
Graduate school		1 (5.3)	0 (0.0)	1 (9.1)	3 (11.1)	0 (0.0)	1 (3.2)		
Employment history (%)		19 (100.0)	12 (100.0)	11 (100.0)	26 (96.3)	11 (100.0)	29 (93.5)		
Current employment (%)		7 (36.8)	4 (33.3)	7 (63.6)	11 (40.7)	9 (81.8)	26 (83.9)		
Marital history (%)		9 (47.4)	6 (50.0)	8 (72.7)	13 (48.1)	10 (90.9)	22 (71.0)		
Divorce history (%)		6 (31.6)	5 (41.7)	2 (18.2)	6 (22.2)	1 (9.1)	3 (9.7)		
Smoking (%)		4 (21.1)	3 (25.0)	4 (36.4)	6 (22.2)	0 (0.0)	1 (3.2)		
Alcohol intake (%)		6 (31.6)	5 (41.7)	4 (36.4)	8 (29.6)	1 (9.1)	18 (58.1)		
Substance use (%)		1 (5.3)	2 (16.7)	0 (0.0)	0 (0.0)	0 (0.0)	0 (0.0)		
Physical disease (%)		15 (78.9)	4 (33.3)	9 (81.8)	15 (55.6)	8 (72.7)	14 (45.2)		
Psychiatric comorbidity (%)		3 (15.8)	2 (16.7)	2 (18.2)	4 (14.8)	0 (0.0)			
Family psychiatric history (%)		8 (42.1)	3 (25.0)	7 (63.6)	8 (29.6)	7 (63.6)	1 (3.2)		
Type, Bipolar I/II		6/13	7/5	4/6					
Bipolarity (%)		18 (94.7)	12 (100.0)	10 (90.9)	5 (18.5)	0 (0.0)	0 (0.0)		
Disease duration, years (*SD*)		12.63 (7.07)	12.25 (13.15)	11.00 (7.46)	7.53 (6.06)	11.64 (9.85)			
QIDS-J, points (*SD*)		14.53 (4.66)	10.83 (3.98)	3.36 (1.77)	12.30 (4.16)	2.91 (1.51)	1.65 (1.70)		
YMRS, points (*SD*)		1.79 (2.36)	12.25 (4.55)	0.00 (0.000)	0.63 (1.39)	0.09 (0.29)	0.23 (0.66)		
STAI, points (*SD*)		115.58(22.13)	97.42 (26.27)	77.91 (21.37)	114.52 (14.84)	73.27 (22.15)	68.32 (10.42)		
STAI-S (state), points (SD)		53.05 (11.87)	43.58 (13.30)	38.55 (10.79)	52.93 (9.31)	34.73 (9.24)	34.55 (6.20)		
STAI-T (trait), points (SD)		62.53 (11.32)	53.83 (14.12)	39.36 (10.90)	61.59 (7.23)	38.55 (14.35)	33.77 (6.35)		
LSAS-J, points (*SD*)		66.11 (36.79)	48.67 (23.94)	54.00 (32.83)	73.11 (28.48)	42.55 (27.85)	21.19 (16.53)		
AQ, points (*SD*)		24.53 (9.30)	21.92 (4.66)	19.45 (5.43)	25.59 (6.00)	17.55 (6.64)	13.16 (5.27)		
IES-R, points (*SD*)		39.53 (16.80)	31.50 (20.70)	26.18 (8.77)	32.85 (14.50)	18.00 (11.65)	4.65 (5.20)		
I ntrusion, points (*SD*)		15.32 (7.29)	10.83 (7.86)	9.91 (6.02)	10.33 (6.17)	8.00 (6.42)	2.26 (3.29)		
Avoidance, points (*SD*)		14.58 (6.19)	12.92 (8.03)	12.91 (3.73)	15.15 (7.22)	7.91 (4.94)	1.87 (2.27)		
Hyperarousal, points (*SD*)		9.63 (6.48)	7.75 (6.10)	3.36 (2.14)	7.37 (5.93)	2.09 (2.23)	0.52 (0.91)		
ADA (%)		13 (68.4)	4 (33.3)	5 (45.5)	15 (55.6)	3 (27.3)	1 (3.2)		

Variables represent mean (SD).

*SD* standard deviation, *QIDS-J* Quick Inventory of Depressive Symptomatology Self-Report Japanese version, *YMRS* Young Mania Rating Scale, *STAI* State-Trait Anxiety Inventory, *LSAS-J* Liebowitz Social Anxiety Scale of Japanese version, *AQ* Autism-Spectrum Quotient, *IES-R* Impact of Event Scale-Revised, *ADA* Anxious-depressive attack.

**Table 2:** Psychiatric comorbidities of patient groups.

	Bipolar disorder			Major depressive disorder	
	Depression	Mania/hypomania	Remission	Depression	Remission
	(*n* = 19)	(*n* = 12)	(*n* = 11)	(*n* = 27)	(*n* = 11)
Psychiatric comorbidity (%)	3(15.8)	2 (16.7)	2 (18.2)	4 (14.8)	0 (0.0)
Alcohol dependence (%)	1 (5.3)	0 (0.0)	1 (9.1)	1 (3.7)	0 (0.0)
Social anxiety disorder (%)	0 (0.0)	1 (8.3)	1 (9.1)	1 (3.7)	0 (0.0)
Panic disorder (%)	0 (0.0)	0 (0.0)	0 (0.0)	1 (3.7)	0 (0.0)
Obsessive–compulsive disorder (%)	1 (5.3)	0 (0.0)	0 (0.0)	0 (0.0)	0 (0.0)
Conversion disorder (%)	0 (0.0)	0 (0.0)	0 (0.0)	1 (3.7)	0 (0.0)
Autism spectrum disorder (%)	1 (5.3)	0 (0.0)	0 (0.0)	0 (0.0)	0 (0.0)
Attention-deficit hyperactivity disorder (%)	0 (0.0)	1 (8.3)	0 (0.0)	0 (0.0)	0 (0.0)

### Classification of life events related to ERPD

Table 3 shows life events related to ERPD for all participants. Of the 23 participants with overlapping ERPD-related events, only one—a patient with BD—reported three ERPD-related events.

**Table 3:** Classification of life events related to ERPD and time-related information of ERPD, based on all participants.

	Bipolar disorder			Major depressive disorder		Healthy subjects
	Depression	Mania/hypomania	Remission	Depression	Remission	
	(*n* = 19)	(*n* = 12)	(*n* = 11)	(*n* = 27)	(*n* = 11)	(*n* = 31)
Classification of life events related to ERPD (%)						
Family problems with no abuse	2 (10.5)	2 (16.7)	2 (18.2)	3 (11.1)	0 (0.0)	1 (3.2)
Separation from close person	2 (10.5)	0 (0.0)	0 (0.0)	2 (7.4)	1 (9.1)	2 (6.5)
Interpersonal-related events	3 (15.8)	1 (8.3)	2 (18.2)	4 (14.8)	3 (27.3)	5 (16.1)
Health-related events	5 (26.3)	1 (8.3)	4 (36.4)	4 (14.8)	3 (27.3)	1 (3.2)
Money-related events	1 (5.3)	1 (8.3)	1 (9.1)	0 (0.0)	0 (0.0)	0 (0.0)
Sex-related events	0 (0.0)	2 (16.7)	0 (0.0)	1 (3.7)	1 (9.1)	1 (3.2)
Change of living conditions	0 (0.0)	1 (8.3)	0 (0.0)	1 (3.7)	2 (18.2)	5 (16.1)
Job-related events	7 (36.8)	5 (41.7)	3 (27.3)	15 (55.6)	5 (45.5)	14 (45.1)
Bullying, neglect	3 (15.8)	2 (16.7)	0 (0.0)	4 (14.8)	0 (0.0)	2 (6.5)
Other events	0 (0.0)	0 (0.0)	1 (9.1)	0 (0.0)	0 (0.0)	1 (3.2)
Overlapping events	4 (21.1)	2 (16.7)	2 (18.2)	7 (25.9)	4 (36.4)	2 (6.5)
Duration of occurrence of events related to ERPD (year)	10.3	17.4	7.4	7	8.5	11.5
Frequency of occurrence of ERPD (%)						
Not at all	3 (15.8)	3 (25.0)	5 (45.5)	2 (7.4)	5 (45.5)	18 (58.1)
Once or twice a month	7 (36.8)	3 (25.0)	2 (18.2)	14 (51.9)	2 (18.2)	10 (32.2)
Once or twice a week	4 (21.1)	2 (16.7)	2 (18.2)	3 (11.1)	1 (9.1)	1 (3.2)
Several times a week	2 (10.5)	2 (16.7)	2 (18.2)	3 (11.1)	1 (9.1)	1 (3.2)
Almost every day	3 (15.8)	1 (8.3)	0 (0.0)	3 (11.1)	2 (18.2)	1 (3.2)
Duration when ERPD occurs (%)						
Less than 1 min	3 (15.8)	1 (8.3)	4 (36.4)	1 (3.7)	4 (36.4)	18 (58.1)
1 min to less than 5 min	3 (15.8)	4 (33.3)	2 (18.2)	10 (37.0)	4 (36.4)	12 (38.7)
5 min to less than 10 min	6 (31.6)	4 (33.3)	2 (18.2)	10 (37.0)	0 (0.0)	1 (3.2)
10 min to less than 20 min	0 (0.0)	0 (0.0)	1 (9.1)	3 (11.1)	1 (9.1)	0 (0.0)
20 min to less than 30 min	1 (5.3)	0 (0.0)	0 (0.0)	0 (0.0)	1 (9.1)	0 (0.0)
30 min to less than 40 min	2 (10.5)	2 (16.7)	2 (18.2)	0 (0.0)	1 (9.1)	0 (0.0)
40 min to less than 50 min	0 (0.0)	0 (0.0)	0 (0.0)	0 (0.0)	0 (0.0)	0 (0.0)
50 min to less than 1 h	1 (5.3)	0 (0.0)	0 (0.0)	1 (3.7)	0 (0.0)	0 (0.0)
More than 1 h	3 (15.8)	1 (8.3)	0 (0.0)	2 (7.4)	0 (0.0)	0 (0.0)

*ERPD* event-related psychological distress.

### Length of occurrence of life events, frequency, and sustained duration of ERPD

Table 3 also shows time-related information for ERPD. Regarding the length of the occurrence of life events related to ERPD, there were no correlations between length and total ERPD-24 scores in patients with MDD and BD and healthy subjects. In terms of each ERPD-composing factor, there was a significant negative correlation between the length and the scores of “self-denial” (*r* = − 0.35, *p* < 0.05) in patients with BD and a significant positive correlation between the length and scores of “mental paralysis” (*r* = 0.44, *p* < 0.05) in healthy subjects. The scores for the other ERPD factors—“feelings of revenge” and “rumination”—did not correlate with length.

Regarding ERPD occurrence frequency, significant positive correlations were found between frequency and the scores for total ERPD-24 (*r* = 0.47, *p* < 0.01), “rumination” (*r* = 0.63, *p* < 0.01), “self-denial” (*r* = 0.47, *p* < 0.01), and “mental paralysis” (*r* = 0.40, *p* < 0.05) in patients with MDD. Among BD patients, there were no positive correlations between frequency and the scores for total ERPD-24 and “self-denial”; meanwhile, frequency demonstrated a significant positive correlation with “rumination” and “mental paralysis” in this group. There were no correlations between frequency and any type of ERPD-24 score in healthy subjects.

In terms of the duration of ERPD, there were no correlations between length and any type of ERPD-24 score in patients with BD or healthy subjects. Conversely, duration was positively correlated with the total ERPD-24 score (*r* = 0.40, *p* < 0.05) and the scores for “rumination” (*r* = 0.38, *p* < 0.05) and “self-denial” (*r* = 0.44, *p* < 0.01) in patients with MDD (there were no correlations between duration and the scores for “feelings of revenge” and “mental paralysis” in this group).

### The severity of ERPD among the groups

**Figure 2:**
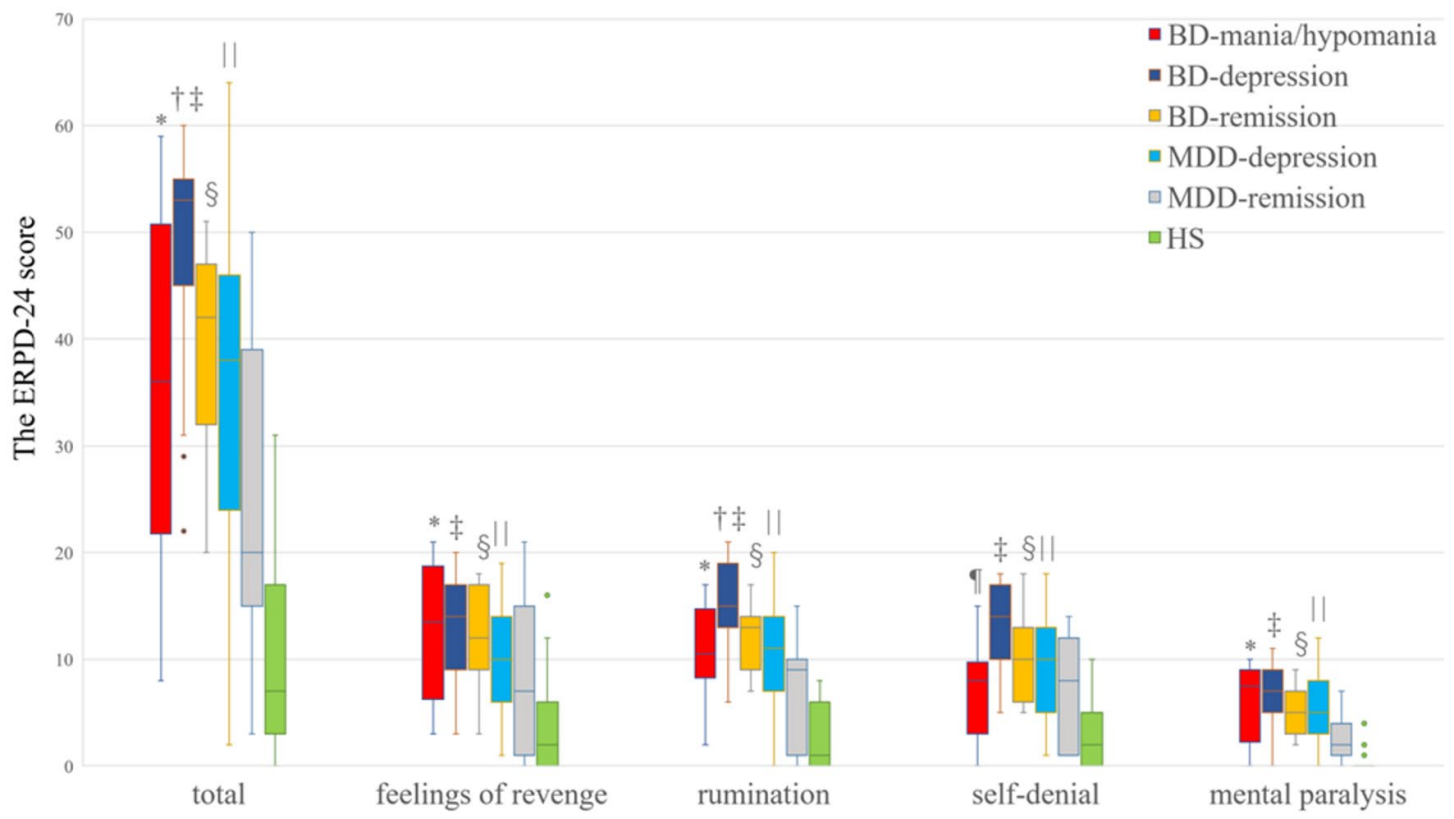
ERPD-24 scores among the 6 groups. ERPD-24 scores (total and sub-categories: feelings of revenge, rumination, self-denial, mental paralysis) for the 6 groups: BD-manic/hypomanic, BD-depression, BD-remission, MDD-depression, MDD-remission, and healthy subjects (HS). The statistical analyses were used with the Kruskal–Wallis test to compare the differences between the groups, followed by the Dunn–Bonferroni adjusted post-hoc test for multiple comparisons. The crossbar of the box indicates the median, the box spans the interquartile range (25–75th percentiles), and the whiskers mark the minimum and maximum values, with outliers identified as individual points. *Comparison between BD-mania/hypomania and HS (*p* < 0.01); †Comparison between BD-depression and MDD-remission (*p* < 0.05); ‡Comparison between BD-depression and HS (*p* < 0.01); §Comparison between BD-remission and HS (*p* < 0.01); ||Comparison between MDD-depression and HS (*p* < 0.01); ¶Comparison between BD-mania/hypomania and BD-depression (*p* < 0.05). *BD* bipolar disorder, *MDD* major depressive disorder.

A test for the normality of the ERPD using the Shapiro–Wilk test did not assume normality. Additionally, the tests for the homogeneity of variances in the ERPD-24 scores were not uniform, except for “self-denial”. Therefore, we used the Kruskal–Wallis test to compare the differences between the groups, followed by the Dunn–Bonferroni adjusted post-hoc test for multiple comparisons. There were significant differences among the six groups in the ERPD-24 total score and the scores for “feelings of revenge”, “rumination”, “self-denial”, and “mental paralysis” (*p* < 0.01). The post-hoc analysis showed significant differences between the groups (Fig. 2). As Fig. 2 outlines, the total score and the scores for “feelings of revenge,” “rumination,” and “mental paralysis” in non-remitted MDD patients and BD patients across different states (i.e., depressive, manic/hypomanic, and remitted) were significantly higher than those in healthy subjects, although those in remitted MDD patients were not significant. The scores for “self-denial” were significantly higher in non-remitted MDD patients and depressive- and remission-state BD patients than in healthy subjects. Conversely, there were no significant differences in the scores for “self-denial” across remitted MDD and manic/hypomanic BD patients compared to healthy subjects.

### Relationship between ERPD and anxiety-related symptoms

Table 4 shows the results for ERPD and anxiety-related psychometrics in patients with MDD and BD. Regarding depression, there were significant positive correlations between the QIDS-J scores and the total ERPD-24 score and the scores for “rumination,” “self-denial,” and “mental paralysis”; the score for “feelings of revenge” was not correlated with the QIDS-J scores in patients with BD (Table 4). Likewise, there were significant positive correlations between the QIDS-J scores and the total score for the ERPD-24 as well as the scores for “self-denial”; the scores for “feelings of revenge”, “rumination”, and “mental paralysis” were not correlated with the QIDS-J scores among healthy subjects (Table 4). Conversely, there were no correlations between the QIDS-J scores and the total score for the ERPD-24 and the scores for “feelings of revenge”, “rumination”, and “self-denial” in patients with MDD, even though only the score for “mental paralysis” was positively correlated with the QIDS-J scores for this group (Table 4).

**Table 4:** Correlations between ERPD and depression/anxiety/trauma-related characteristics.

	QIDS-J	STAI-S (state)	STAI-T (trait)	LSAS-J	IES-R			
					Total	Intrusion	Avoidance	Hyperarousal
Total								
ERPD-24	0.609**	0.585**	0.698**	0.599**	0.863**	0.790**	0.755**	0.735**
Feelings of revenge	0.430**	0.377**	0.506**	0.440**	0.660**	0.577**	0.619**	0.540**
Rumination	0.598**	0.623**	0.691**	0.611**	0.852**	0.794**	0.730**	0.729**
Self-denial	0.493**	0.448**	0.569**	0.477**	0.664**	0.641**	0.568**	0.543**
Mental paralysis	0.623**	0.613**	0.673**	0.551**	0.834**	0.738**	0.706**	0.777**
Bipolar disorder								
ERPD-24	0.445**	0.425**	0.494**	0.414**	0.669**	0.613**	0.523**	0.600**
Feelings of revenge	0.199	0.144	0.210	0.181	0.432**	0.340*	0.447**	0.343*
Rumination	0.476**	0.559**	0.559**	0.494**	0.684**	0.606**	0.543**	0.631**
Self-denial	0.342*	0.214	0.318*	0.306*	0.352*	0.402**	0.168	0.329*
Mental paralysis	0.374*	0.468**	0.493**	0.299	0.651**	0.593**	0.485**	0.612**
Major depressive disorder								
ERPD-24	0.243	0.585**	0.624**	0.455**	0.897**	0.766**	0.638**	0.730**
Feelings of revenge	0.102	0.320	0.439**	0.284	0.530**	0.393*	0.419**	0.445**
Rumination	0.237	0.583**	0.540**	0.435**	0.860**	0.802**	0.571**	0.677**
Self-denial	0.115	0.422**	0.486**	0.302	0.713**	0.647**	0.536**	0.499**
Mental paralysis	0.413**	0.599**	0.550**	0.480**	0.803**	0.614**	0.522**	0.799**
Healthy control								
ERPD-24	0.363*	0.214	0.500**	0.552**	0.720**	0.660**	0.542**	0.374*
Feelings of revenge	0.110	0.052	0.184	0.416*	0.596**	0.534**	0.465**	0.317
Rumination	0.212	0.257	0.603**	0.615**	0.685**	0.626**	0.488**	0.436*
Self-denial	0.523**	0.182	0.380*	0.269	0.291	0.305	0.209	0.037
Mental paralysis	0.247	0.260	0.467**	0.233	0.659**	0.530**	0.550**	0.482**

*p < 0.05, ** p < 0.01.

*ERPD* event-related psychological distress, *QIDS-J* Quick Inventory of Depressive Symptomatology Self-Report Japanese version, *STAI* State-Trait Anxiety Inventory, *LSAS-J* Liebowitz Social Anxiety Scale of Japanese version, *IES-R* Impact of Event Scale-Revised.

Regarding anxiety-related symptoms, the scores for both the STAI-state and STAI-trait were significantly positively correlated with the total score for the ERPD-24 and the scores for “rumination” and “mental paralysis” in the BD and MDD groups (Table 4). Among healthy subjects, the positive correlations between the scores for the STAI trait were significantly positively correlated with those for the ERPD-24, although there were no correlations between the scores for the STAI-state and the ERPD-24 (Table 4). The scores for the LSAS were significantly positively correlated with the total score for the ERPD-24 and the score for “rumination” in all three groups (Table 4). In terms of ADA, among the BD and MDD patient groups, all ERPD-24 scores were significantly higher in those with ADA than in those without ADA (Table 5).

**Table 5:** ERPD-24 scores in BD and MDD patients with or without ADA.

All patients	Without ADA	With ADA	*t*	*p*	95% CI^a^
80	40	40			
Total ERPD-24	32.63 (15.036)	42.40 (13.821)	− 3.027	0.003	− 16.204, − 3.346
Feelings of revenge	11.00 (5.666)	11.38 (5.564)	− 0.299	0.766	− 2.875, 2.125
Rumination	9.10 (4.684)	13.90 (4.634)	− 4.607	0.000	− 6.874, − 2.726
Self-denial	8.00 (4.946)	11.10 (4.754)	− 2.858	0.005	− 5.260, − 0.940
Mental paralysis	4.53 (3.146)	6.03 (3.034)	− 2.171	0.033	− 2.876, − 0.124

Variables represent mean (standard deviation: SD).

*ERPD* event-related psychological distress, *ADA* Anxious-depressive attack.

^a^Statistical analysis was performed by Student’s t-test.

### Relationship between the other psychometrics and ERPD

Regarding the relationship between the scores for the ERPD-24 and those for the IES-R, Table 4 shows that almost all scores for the ERPD-24 (i.e., the scores for “feelings of revenge,” “rumination,” “self-denial,” and “mental paralysis”) were significantly positively correlated with those for the IES-R (including total, intrusion, avoidance, and hyperarousal) in patients with MDD, BD, and healthy subjects; however, there was no relationship between the scores for “self-denial” and avoidance in BD patients and the scores for “self-denial” and the IES-R total and sub-component scores.

The ERPD-24 total score and the scores for “rumination” and “mental paralysis” in MDD patients with bipolarity were significantly higher than in those without bipolarity.

Of the total patients, 11 patients (five with MDD and six with BD) with autistic traits were included in this study. A BD patient with autism spectrum disorder scored 31 on the AQ-J (notably, this is < 33). There were no significant differences in the total ERPD-24 score and the ERPD-24 scores for the four comprising factors between patients with MDD and BD with and without autistic traits.

## Discussion

This study had three main findings. First, non-remitted MDD patients and BD patients regardless of remission/non-remission (i.e., manic/hypomanic and depressive state), presented more severe ERPD associated with life events under the threshold for traumatic experiences (given by diagnostic criteria for PTSD) than healthy subjects. Second, this study presents the details of ERPD, including temporal information. Third, anxiety-related symptoms, including social phobia and ADA, could be associated with ERPD not only in patients with BD and MDD, but also in healthy subjects.

This is the first study to present the details of ERPD in patients with BD, MDD, and healthy subjects. Regarding the relationship between the duration of occurrence of ERPD-related events and severity, length only correlated with ERPD severity under “self-denial” in patients with BD and “mental paralysis” in healthy subjects. According to similar previous studies on mood disorders^28^ and PTSD^29^, the length of occurrence of stressful events weakly influences the severity and, relatedly, prognosis of these diseases. Adverse childhood experiences have been found to have a prolonged negative influence on various psychiatric disorders in adults^30–33^. Moreover, given that various forms of cognitive dysfunction exist among remitted patients with BD^34^, even if BD patients experience symptomatic remission, they may be likely to have or ruminate on feelings of self-denial related not only to their past but also to more recent life events. As an ERPD-composing factor, self-denial might lead a patient to be more vulnerable to recurrence or relapse because the DSM-5 criteria for a major depressive episode in BD include “feeling worthless or excessive/inappropriate guilt”; this is similar to thoughts and feelings of self-denial. This study adds to the existing research on this topic by revealing that the severity of ERPD may be slightly linked to the duration of occurrence of an ERPD-related event.

Additionally, regarding the length of occurrence of life events in ERPD, the present study shows a significant positive correlation between the length and scores of “mental paralysis” only in healthy people. According to the ERPD-24 scale (as seen in the Supplemental File), “mental paralysis” as an ERPD-composing factor consists of four experiences: heaviness, fogginess, a loss of concentration, and a loss of will or intention when recalling ERPD-related events. Conversely, there are no correlations between the length of the occurrence of ERPD-related life events and the scores of “mental paralysis” among the BD and MDD patient groups. Accumulating evidence reports that BD and MDD patients, regardless of symptomatic remission/non-remission, suffer from cognitive impairment including concentration^35,36^. Therefore, cognitive impairment may influence “mental paralysis” comprising initiative and concentration in BD and MDD patients. Our findings suggest that having more past experiences leading to episodic memories may have a stronger impact on ERPD, especially among healthy people with mental paralysis. To further clarify this finding, further studies with larger sample sizes are needed.

Conversely, this study reveals that the frequency of occurrence and duration of ERPD are positively correlated with the severity of ERPD in patients with MDD, although they are not correlated in healthy subjects. According to previous reports, depressed individuals have difficulty suppressing involuntary thoughts^37^ and autobiographical memories^38^; these neuropsychological phenomena may overlap with ERPD. To improve understandings of and treatments for MDD, researchers and physicians should carefully focus on ERPD and related psychological features in depressed patients.

No correlations between the frequency and duration of the occurrence of ERPD and the severity of ERPD were found among BD patients; however, “rumination” and “mental paralysis” were partly positively correlated with frequency in this group. Given that various psychological conditions occur in BD patients with depression, mania/hypomania, and euthymia, the components of ERPD may vary among patients with BD. Therefore, the small sample size of the present study may have not be able to clarify the correlations between the frequency and duration of the occurrence and the severity of ERPD. Further studies with a large sample size and observational cohort design are needed to clarify the association between ERPD and mood states in BD.

This study found that, compared to healthy subjects, patients with non-remitted MDD, and BD regardless of remission/non-remission, experience more psychological distress associated with past events in daily life (i.e., they experience ERPD, consisting of feelings of revenge, rumination, self-denial, and mental paralysis), even if these events are necessarily under the traumatic experience threshold set by the diagnostic criteria for PTSD. These findings are mainly consistent with our hypothesis, although the severity of ERPD in remitted BD patients was higher than that in healthy people. Regarding MDD, the present findings are consistent with our previous study’s^4^ observation that psychological distress is associated with disease-onset events in daily life in non-remitted MDD patients (this study used the IES-R). Additionally, this study contributes to existing knowledge about psychopathology in mood disorders by revealing that MDD patients currently in a depressive state (i.e., non-remission) experience more ERPD characterized by self-denial, rumination, mental paralysis, and feelings of revenge in relation to past life events than healthy subjects. Regarding feelings of revenge, several previous studies report that patients with depression demonstrate trait anger/hostility^39,40^ and irritability^41^. Accordingly, our findings imply that depressed patients may have ambivalent feelings, such as feelings of revenge and self-denial, when they recall their past life events and experience ERPD.

Conversely, patients with BD, regardless of whether they were in a remission or non-remission state, experienced severe ERPD in this study. This finding is somewhat surprising: our previous study reported that euthymic BD patients have milder ERPD than depressive and manic/hypomanic (i.e., non-remitted) ones^5^. This may have occurred because we asked participants about onset-related and onset-unrelated past events at the same time during face-to-face interviews in this earlier study. Ultimately, 70.9% (n = 56) of patients answered that they felt that they had experienced both kinds of events, and 19.6% (n = 11) responded that the events were the same^5^. The ERPD-24-based method used in the current study may more accurately identify ERPD than the method used in our previous study. However, further studies with larger sample sizes are needed to reveal the association between ERPD and mood states in BD patients.

This study revealed a strong association between anxiety-related symptoms and ERPD in both BD and MDD patients and healthy subjects. Turner et al. reported that intrusive thoughts characterized by worry, including those related to obsessive–compulsive and anxiety disorders, may occur more frequently in people with mood disorders^42^. In addition, previous studies have suggested a relationship between interpersonal problems and mental problems, including social anxiety, rejection sensitivity, and depression^43,44^. These findings support those of the present study regarding the relationship between ERPD. Furthermore, among patients with MDD and BD, the total ERPD scores of patients with ADA were higher than those of patients without ADA. According to Kaiya’s proposal of the concept of ADA^11^, ADA is similar to panic attacks in the brain; notably, both are accompanied by intrusive thoughts and rumination. Interestingly, ADA level has been associated with rejection sensitivity^45^. These findings suggest that ERPD may be especially associated with social anxiety and rejection sensitivity, and that severe ERPD may occur with ADA in patients with mood disorders and other psychiatric diseases. In addition, this study also found that a relationship exists between ERPD and anxiety. Almost all healthy people experience anxiety or fear in daily life^42^, although previous studies report that the degree of anxiety is lower in those with anxiety disorders, especially those with generalized anxiety disorder^41,46^. Therefore, once healthy subjects with a threshold clinical mental illness experience anxiety, they may experience ERPD associated with past events. Further studies are needed to reveal the relationship between ERPD and anxiety-related assessments.

Regarding depression, the severity of depression in patients with MDD was not correlated with the ERPD scores in this study, except regarding mental paralysis; however, positive correlations were found between depression severity in patients with BD and healthy subjects. This finding differs from those of our previous studies that showed the relationships between the severity of depression and that of ERPD in patients with MDD and BD^4,5^, perhaps because we used different assessment tools to measure depression severity. The QIDS-J includes symptoms of hypersomnia and increased appetite, which are not considered by the Hamilton Depression Rating Scale (HAMD)^47^. In addition, there is a difference in raters: the QIDS-J is a self-administered scale; the HAMD was assessed by physicians or researchers Nevertheless, since the relationship between the QIDS-J and HAMD is obvious, further studies with a large sample size are needed to confirm this.

The concept of ERPD can provide new insights and perspectives from the clinical presentation to the pathophysiology in patients with MDD and BD. In clinical insights, considering the existence of ERPD rather than focusing on well-known terminological symptoms such as depressed mood, could promote a better understanding of the complex and various psychiatric pathology and behaviours in patients with MDD and BD. For instance, regarding hopelessness and demoralization in suicidal behaviour, as one of the most important psychiatric conditions^48^, the insights of ERPD might help researchers resolve the pathophysiology with potential new approaches, including considering neuroinflammatory abnormalities such as the kynurenine pathway^49,50^ in patients with MDD and BD. The presentation of ERPD could contribute to further studies that can challenge the current understanding and the management of mood disorders.

This study had some limitations. First, this study only examined six groups, which meant that it was likely to yield complex results. Second, the study may have involved subject recall bias. Third, the sample size was small. Considering BD and MDD patients were divided into three (BD mania/hypomania, BD depression, and BD remission) and two (MDD depression and remission) subgroups to compare them with healthy subjects, the implementation of a large sample size could further clarify the differences between the severity and contents of ERPD among the groups. Finally, this was a cross-sectional study, and it did not identify or investigate changes in ERPD among the participants. To overcome these problems, a prospective study with a larger sample size for each group is needed.

In conclusion, this study demonstrated that patients with non-remitted MDD, and BD regardless of remission/non-remission, more frequently experience severe ERPD associated with life events under the threshold for traumatic experiences than healthy subjects. Considering that depressive disorders, in which one-third of patients do not reach remission^51^, have the highest burden on global health of all psychiatric diseases^52^, and that almost all patients with BD need long-term treatment^53^, many patients with mood disorders can be thought to be suffering from ERPD. ERPD can provide psychiatrists with new insights into the clinical features of mood disorders; in particular, focusing on ERPD can improve their understanding of psychopathology in patients with MDD and BD. Furthermore, we found that ERPD is associated with anxiety. These findings are useful because they may encourage researchers and physicians to focus on such patients with ERPD, who may otherwise have been overlooked.

### Supplementary Information

Supplementary Information.

## Data Availability

The datasets generated and/or analyzed during the current study are available from the corresponding author upon reasonable request.

## References

[1] American Psychiatric Association. *Diagnostic & Statistical Manual of Mental Disorders*, 5th ed*.* (2013).

[2] Kendler KS, Gardner CO (2010). Dependent stressful life events and prior depressive episodes in the prediction of major depression: The problem of causal inference in psychiatric epidemiology. Arch. Gen. Psychiatry.

[3] Lex C, Bäzner E, Meyer TD (2017). Does stress play a significant role in bipolar disorder? A meta-analysis. J. Affect. Disord..

[4] Kimura A, Hashimoto T, Niitsu T, Iyo M (2015). Presence of psychological distress symptoms associated with onset-related life events in patients with treatment-refractory depression. J. Affect. Disord..

[5] Sato, A., Hashimoto, T., Kimura, A., Niitsu, T. & Iyo, M. Psychological distress symptoms associated with life events in patients with bipolar disorder: A cross-sectional study. *Front. Psychiatry.* **9**, (2018). 10.3389/fpsyt.2018.00200 PMC5974931 29875708

[6] WHO. *International Statistical Classification of Diseases and Related Health Problems,* 11th revision*.* (2022).

[7] Seki, R. *et al.* ERPD-24. *Chiba University.* https://www.m.chiba-u.ac.jp/class/psy3/education/erpd24.html (accessed 6 May 2024).

[8] Seki R (2021). Identification of psychological features and development of an assessment tool for event-related psychological distress after experiencing non-traumatic stressful events. PLoS One.

[9] Watkins ER (2011). Rumination-focused cognitive-behavioural therapy for residual depression: Phase II randomised controlled trial. Br. J. Psychiatry.

[10] Bell IH (2022). The effect of psychological treatment on repetitive negative thinking in youth depression and anxiety: A meta-analysis and meta-regression. Psychol. Med..

[11] Kaiya H (2017). Distinctive clinical features of “Anxious-Depressive Attack”. Anxiety Disord. Res..

[12] Ibrahim K (2019). Anger rumination is associated with restricted and repetitive behaviors in children with autism spectrum disorder. J. Autism Dev. Disord..

[13] Otsubo T (2005). Reliability and validity of Japanese version of the mini-international neuropsychiatric interview. Psychiatry Clin. Neurosci..

[14] Sheehan, D. *et al.* The Mini-International Neuropsychiatric Interview (MINI): The development and validation of a structured diagnostic psychiatric interview for DSM-IV and ICD-10. *J. Clin. Psychiatry.* **59**(Suppl 2) (1998). 9881538

[15] Rush AJ (2003). The 16-item Quick inventory of depressive symptomatology (QIDS), clinician rating (QIDS-C), and self-report (QIDS-SR): A psychometric evaluation in patients with chronic major depression. Biol. Psychiatry.

[16] Fujisawa D (2010). Cross-cultural adaptation of the quick inventory of depressive symptomatology-self report (QIDS-SR-J). Jpn. J. Stress Sci..

[17] Young RC, Biggs JT, Ziegler VE, Meyer DA (1978). A rating scale for mania: Reliability, validity and sensitivity. Br. J. Psychiatry.

[18] Brugha TS, Cragg D (1990). The list of threatening experiences: The reliability and validity of a brief life events questionnaire. Acta Psychiatr. Scand..

[19] Spielberger, C., Gorsuch, R., Lushene, R., Vagg, P. R. & Jacobs, G. *Manual for the State-Trait Anxiety Inventory (Form Y1–Y2)*, vol. IV (Consulting Psychologists Press, 1983).

[20] Mizuguchi K, Simonaka J, Nakasato K (1970). Japanese Version STAI Manual.

[21] Asakura, S. *et al.* Reliability and validity of the Japanese version of the Liebowitz social anxiety scale. *Seishin Igaku Clin. Psychiatry* **44,** (2002).

[22] Weiss, D. S. & Marmar, C. R. The impact of event scale-revised. In *Assessing Psychological Trauma and PTSD: A Handbook for Practitioners.* 399–451 (Guilford Press, 1997).

[23] Asukai N (2002). Reliability and validity of the Japanese-language version of the impact of event scale-revised (IES-R-J): Four studies of different traumatic events. J. Nerv. Ment. Dis..

[24] Baron-Cohen S, Wheelwright S, Skinner R, Martin J, Clubley E (2001). The autism-spectrum quotient (AQ): Evidence from Asperger syndrome/ high-functioning autism, males and females, scientists and mathematicians. J. Autism Dev. Disord..

[25] Wakabayashi A, Tojo Y, Baron-Cohen S, Wheelwright S (2004). The autism-spectrum quotient (AQ) Japanese version: Evidence from high-functioning clinical group and normal adults. Jpn. J. Psychol..

[26] Ghaemi SN, Ko JY, Goodwin FK (2002). ‘Cade’s disease’ and beyond: Misdiagnosis, antidepressant use, and a proposed definition for bipolar spectrum disorder. Can. J. Psychiatry..

[27] Angst J (2003). Toward a re-definition of subthreshold bipolarity: Epidemiology and proposed criteria for bipolar-II, minor bipolar disorders and hypomania. J. Affect. Disord..

[28] Roca M (2013). Stressful life events severity in patients with first and recurrent depressive episodes. Soc. Psychiatry Psychiatr. Epidemiol..

[29] Bryant RA, O’Donnell ML, Creamer M, McFarlane AC, Silove D (2013). A multisite analysis of the fluctuating course of posttraumatic stress disorder. JAMA Psychiatry.

[30] Park YM, Shekhtman T, Kelsoe JR (2020). Effect of the type and number of adverse childhood experiences and the timing of adverse experiences on clinical outcomes in individuals with bipolar disorder. Brain Sci..

[31] LeMoult J (2020). Meta-analysis: Exposure to early life stress and risk for depression in childhood and adolescence. J. Am. Acad. Child Adolesc. Psychiatry.

[32] Daniel WH, Joan K (2018). Adverse childhood experiences in children with autism spectrum disorder. Curr. Opin. Psychiatry.

[33] Crouch E, Radcliff E, Bennett KJ, Brown MJ, Hung P (2021). Examining the relationship between adverse childhood experiences and ADHD diagnosis and severity. Acad. Pediatr..

[34] Solé B (2017). Cognitive impairment in bipolar disorder: Treatment and prevention strategies. Int. J. Neuropsychopharmacol..

[35] Little B, Anwyll M, Norsworthy L, Corbett L, Schultz-Froggatt M, Gallagher P (2024). Processing speed and sustained attention in bipolar disorder and major depressive disorder: A systematic review and meta-analysis. Bipolar Disord..

[36] Semkovska M (2019). Cognitive function following a major depressive episode: A systematic review and meta-analysis. Lancet Psychiatry..

[37] Wenzlaff RM, Wegner DM (2000). Thought suppression. Annu. Rev. Psychol..

[38] Watson LA, Berntsen D, Kuyken W, Watkins ER (2012). The characteristics of involuntary and voluntary autobiographical memories in depressed and never depressed individuals. Conscious. Cogn..

[39] de Bles NJ (2019). Trait anger and anger attacks in relation to depressive and anxiety disorders. J. Affect. Disord..

[40] Fisher LB (2015). The role of anger/hostility in treatment-resistant depression: A secondary analysis from the ADAPT-A study. J. Nerv. Ment. Dis..

[41] Fava M (2009). The importance of irritability as a symptom of major depressive disorder: Results from the national comorbidity survey replication. Mol. Psychiatry 2010 158.

[42] Dupuy JB, Beaudoin S, Rhéaume J, Ladouceur R, Dugas MJ (2001). Worry: Daily self-report in clinical and non-clinical populations. Behav. Res. Ther..

[43] Normansell KM, Wisco BE (2017). Negative interpretation bias as a mechanism of the relationship between rejection sensitivity and depressive symptoms. Cogn. Emot..

[44] Gao S, Assink M, Cipriani A, Lin K (2017). Associations between rejection sensitivity and mental health outcomes: A meta-analytic review. Clin. Psychol. Rev..

[45] Kaiya H (2020). Effects of rejection sensitivity on the development of anxious-depressive attack in patients with social anxiety disorder. Anxiety Disord. Res..

[46] Craske MG, Rapee RM, Jackel L, Barlow DH (1989). Qualitative dimensions of worry in DSM-III-R generalized anxiety disorder subjects and nonanxious controls. Behav. Res. Ther..

[47] Hamilton M (1960). A rating scale for depression. J. Neurol. Neurosurg. Psychiatry.

[48] Costanza D (2022). Demoralization in suicide: A systematic review. J. Psychosom. Res..

[49] Serafini G (2017). Abnormalities in kynurenine pathway metabolism in treatment-resistant depression and suicidality: A systematic review. CNS Neurol. Disord. Drug Targets..

[50] Savitz J (2020). The kynurenine pathway: A finger in every pie. Mol. Psychiatry..

[51] Rush AJ (2006). Acute and longer-term outcomes in depressed outpatients requiring one or several treatment steps: a STAR*D report. Am. J. Psychiatry..

[52] GBD 2019 Diseases and Injuries Collaborators. Global burden of 369 diseases and injuries in 204 countries and territories, 1990–2019: A systematic analysis for the Global Burden of Disease Study 2019. *Lancet.* **396,** 1204–1222 (2020). 10.1016/S0140-6736(20)30925-9 PMC7567026 33069326

[53] Yatham LN (2018). Canadian Network for Mood and Anxiety Treatments (CANMAT) and International Society for Bipolar Disorders (ISBD) 2018 guidelines for the management of patients with bipolar disorder. Bipolar Disord..

