# Supplementary material for: Evaluating psychological distress associated with life events under the traumatic experience threshold in patients with major depressive and bipolar disorder
Source: Sci Rep. 2024 Jul 15;14:16264. doi: 10.1038/s41598-024-67101-x (PMC11250807; doi:10.1038/s41598-024-67101-x)
Supplement: Supplementary file 1 — Supplementary Information. [file 41598_2024_67101_MOESM1_ESM.docx]

| **ERPD-24 scale Ver.1.0**Name　　　　　 （Male・Female・Other）Age Date | | | | | |
| --- | --- | --- | --- | --- | --- |
| Have you ever undergone any of the following experiences? Have you ever seen or been directly involved with a family member or close friend who underwent any of the following experiences?  ・Actual or threatened events (e.g., accidents, dangerous fires, disasters, wars, unnatural deaths), excluding death by disease ・Serious injury ・Physical assault, sexual violence and abuse ・Terrorism or kidnapping/abduction  **□**No　**□**Yes　**（If you choose「No」, please continue to the end.）** | | | | | |
| Even if your experiences are not life-threatening, as illustrated in the above examples, people encounter events which are painful or make us feel uncomfortable in our daily lives. This questionnaire seeks details about events **which you experienced more than 1 month prior,** which still cause you to experience unpleasant memories and feelings. Of those events, please list **one that you still recall most often.**  (If you do not have one, please write “None.” The remaining answers may be left blank.) | | | | | |
|  | | | | | |
| When did this event occur?　　 About（　　 ）Y（　　 ）M ago　（The month field can be left blank） | | | | | |
| In the past month, how often have you recalled the event? (please choose one) **□** 0, Not at all; **□** 1, Once or twice; **□** 2, Once or twice a week; **□** 3, Several times a week;  **□** 4, Almost every day | | | | | |
| How long did you continue to remember the event? (please choose one)  **□** 0, less than 1 minute; **□** 1, 1 minute to less than 5 minutes; **□** 2, 5 minutes to less than 10 minutes; **□** 3, 10 minutes to less than 20 minutes; **□** 4, 20 minutes to less than 30 minutes;  **□** 5, 30 minutes to less than 40 minutes; **□** 6, 40 minutes to less than 50 minutes;  **□** 7, 50 minutes to less than 1 hour;　**□** 8, more than 1 hour (Please elaborate if it is 1 hour or more：（　　）hours） | | | | | |
| Please read the following questions and circle the box that is most applicable to the time(s) you thought of such event **in the last week, including today**. Please note that there three pages of questions. （When unsure of your response, please do not think excessively about it. Instead, select the one that you consider closest to your experience). | | | | | |
|  | | Not at all | Not so much | Somewhat so | Very much so |
| 1 | I feel sick when I recall it. |  |  |  |  |
| 2 | Even if I do not want to think about it, I cannot stop myself. |  |  |  |  |
| 3 | I blame myself when I recall it. |  |  |  |  |
|  | | 0.  Not at all | 1.  Not so much | 2.  Somewhat so | 3.  Very much so |
| 4 | I feel that it has ruined my life. |  |  |  |  |
| 5 | My body feels heavy when I recall it. |  |  |  |  |
| 6 | I feel sorry for the people around me (e.g., family members, acquaintances, work colleagues, and/or classmates) when I recall it. |  |  |  |  |
| 7 | I feel heaviness in my chest when I recall it. |  |  |  |  |
| 8 | I wish that the person who caused it, or the cause itself, did not exist. |  |  |  |  |
| 9 | I feel as though I’m a worthless person when I recall it. |  |  |  |  |
| 10 | I feel sad when I recall it. |  |  |  |  |
| 11 | I feel angry when I recall it. |  |  |  |  |
| 12 | I do not believe that I handled it well. |  |  |  |  |
| 13 | I become unable to concentrate when I recall it. |  |  |  |  |
| 14 | I cannot stop thinking about various scenes from it. |  |  |  |  |
|  |  | 0.  Not at all | 1.  Not so much | 2.  Somewhat so | 3.  Very much so |
| 15 | I feel like getting even when I recall it. |  |  |  |  |
| 16 | I feel regret when I recall it. |  |  |  |  |
| 17 | I feel irritated when I recall it. |  |  |  |  |
| 18 | I lose my will to do anything when I recall it. |  |  |  |  |
| 19 | I feel fearful when I recall it. |  |  |  |  |
| 20 | I feel miserable when I recall it. |  |  |  |  |
| 21 | I feel hatred when I recall the cause of it. |  |  |  |  |
| 22 | I feel depressed when I recall it. |  |  |  |  |
| 23 | My head feels foggy when I recall it. |  |  |  |  |
| 24 | I feel as if I’m suffocating when I recall it. |  |  |  |  |

**Event-Related Psychological Distress Scale- 24 (ERPD-24) Version 1.0**

- **What is ERPD?**

**Event-related psychological distress (ERPD)** comprises psychological phenomena such as flashbacks, resentment, and regret related to events considered more mundane, such as interpersonal problems (at home, work, etc.) and financial difficulties, which fall short of the traumatic experiences listed in the diagnostic criteria for post-traumatic stress disorder (PTSD).

This is a self-administered scale to assess the severity of the subject's ERPD in the week prior to and including the date of their response to the questionnaire.

- **Instructions**

First, trauma survivors are excluded using the diagnostic criteria for PTSD (DSM-5 and ICD-11) as described in the instructions. Second, the respondent is asked to fill in the event(s) which still cause them to experience unpleasant memories and feelings and accordingly provide their answers for each item.

- **Scoring method**

This scale is a four-point Likert scale. Scores of 0–3 for each option should be totaled and the score for the entire scale or for each subscale should be calculated. The higher the score of each scale, the higher the severity of ERPD.

- **Subscale components**

Feelings of revenge （7 items）；1,4,8,11,15,17,21

Rumination （7 items）；2,7,10,14,19,22,24

Self-denial　 （6 items）；3,6,9,12,16,20

Mental paralysis （4 items）；5,13,18,23

- **Internal consistency**

**Cronbach’s alpha (*α*)**: Total (*α* = .91), Feelings of revenge (*α* = .88), Rumination (*α* = .85), Self-denial (*α* = .82), Mental paralysis (*α* = .82)

The sample consisted of 747 subjects (373 males, 374 females), aged 20 to 64 years (M = 41.2 years, SD = 12.6 years).

- **Reference**

Seki R, Hashimoto T, Tanaka M, Ishii H, Ogawa M, Sato A, Kimura A, Shiina A, Nakazato M, Iyo M. Identification of psychological features and development of an assessment tool for event-related psychological distress after experiencing non-traumatic stressful events. PLoS One. 2021 Mar 31;16(3):e0249126. doi: 10.1371/journal.pone.0249126. PMID: 33788874; PMCID: PMC8011732.

- Please feel free to use this scale for medical treatment, research, or surveys. However, please refrain from altering, posting, or reprinting the above article in publications without obtaining permission. Please cite the above article (Seki R, Hashimoto T et al., PLoS One. 2021 Mar 31;16(3): e0249126.) in the appropriate manner.

**Contact**:

Tasuku Hashimoto M.D., Ph.D.

Department of Psychiatry

School of Medicine, International University of Health and Welfare (Narita, Japan)

Email: [t.hashimoto1109@gmail.com](mailto:t.hashimoto1109@gmail.com), [t-hashimoto@faculty.chiba-u.jp](mailto:t-hashimoto@faculty.chiba-u.jp), thashimoto@iuhw.ac.jp
